# Real-World Utilization, Barriers, and Factors Associated With the Targeted Treatment of Metastatic Colorectal Cancer Patients in China: A Multi-Center, Hospital-Based Survey Study

**DOI:** 10.3389/ijph.2023.1606091

**Published:** 2023-07-03

**Authors:** Yin Liu, Xi Zhang, Hui-Fang Xu, Ji-Hai Shi, Yu-Qian Zhao, Ling-Bin Du, Yun-Yong Liu, Wen-Jun Wang, He-Lu Cao, Li Ma, Juan-Xiu Huang, Ji Cao, Li Li, Yan-Ping Fan, Xiao-Fen Gu, Chang-Yan Feng, Qian Zhu, Xiao-Hui Wang, Jing-Chang Du, Jian-Gong Zhang, Shao-Kai Zhang, You-Lin Qiao

**Affiliations:** ^1^ Department of Cancer Epidemiology, Affiliated Cancer Hospital of Zhengzhou University, Henan Cancer Hospital, Henan Engineering Research Center of Cancer Prevention and Control, Henan International Joint Laboratory of Cancer Prevention, Zhengzhou, China; ^2^ Key Laboratory of Carcinogenesis and Translational Research (Ministry of Education/Beijing), Beijing Office for Cancer Prevention and Control, Peking University Cancer Hospital and Institute, Beijing, China; ^3^ The Clinical Epidemiology of Research Center, Department of Dermatological, The First Affiliated Hospital of Baotou Medical College, Baotou, China; ^4^ Sichuan Cancer Hospital and Institute, Sichuan Cancer Center, School of Medicine, University of Electronic Science and Technology of China, Chengdu, China; ^5^ Department of Cancer Prevention, The Cancer Hospital of the University of Chinese Academy of Sciences, Zhejiang Cancer Hospital, Hangzhou, China; ^6^ Liaoning Office for Cancer Control and Research, Cancer Hospital of China Medical University, Liaoning Cancer Hospital and Institute, Shenyang, China; ^7^ School of Nursing, Jining Medical University, Jining, China; ^8^ Department of Preventive Health, Xinxiang Central Hospital, Xinxiang, China; ^9^ Public Health School, Dalian Medical University, Dalian, China; ^10^ Department of Gastroenterology, Wuzhou Red Cross Hospital, Wuzhou, China; ^11^ Department of Cancer Prevention and Control Office, The First Affiliated Hospital of Guangxi Medical University, Nanning, Guangxi, China; ^12^ Department of Clinical Research, The First Affiliated Hospital, Jinan University, Guangzhou, China; ^13^ State Key Laboratory of Oncology in South China, Collaborative Innovation Center for Cancer Medicine, Sun Yat-sen University Cancer Center, Guangzhou, China; ^14^ Department of Student Affairs, Affiliated Tumor Hospital, Xinjiang Medical University, Ürümqi, Xinjiang, China; ^15^ Chongqing Key Laboratory of Translational Research for Cancer Metastasis and Individualized Treatment, Chongqing University Cancer Hospital, Chongqing, China; ^16^ School of Public Health and Management, Chongqing Medical University, Chongqing, China; ^17^ Department of Public Health, Gansu Provincial Cancer Hospital, Lanzhou, China; ^18^ School of Public Health, Chengdu Medical College, Chengdu, China; ^19^ Center for Global Health, School of Population Medicine and Public Health, Chinese Academy of Medical Sciences and Peking Union Medical College, Beijing, China

**Keywords:** associated factors, utilization, barriers, metastatic colorectal cancer, targeted treatment

## Abstract

**Objectives:** To explore the utilization, barriers, and factors associated with the targeted treatment of Chinese metastatic colorectal cancer (mCRC) patients.

**Methods:** A total of 1,688 mCRC patients from 19 hospitals in 14 cities were enrolled from March 2020 to March 2021 using stratified, multistage cluster sampling. The use of targeted therapy and any barriers patients experienced were collected. Logistic regression analyses were conducted to identify the factors associated with initiating targeted treatment.

**Results:** About 51.6% of the patients initiated targeted therapy, of whom 44.5%, 20.2%, and 35.2% started first-, second-, and third-line treatment, respectively. The most reported barriers were high medical costs and a lack of belief in the efficacy of targeted therapy. Patients treated in the general hospital, diagnosed at an older age, less educated, and who had a lower family income, no medical insurance, poor health-related quality of life, metastasis outside the liver/lung or systemic metastasis, a shorter duration of mCRC were less likely to initiate targeted therapy.

**Conclusion:** Reduced medical costs and interventional education to improve public awareness could facilitate the use of targeted treatment for mCRC.

## Introduction

Colorectal cancer (CRC) is the most common type of gastrointestinal cancer worldwide and the third leading cause of cancer-related death, resulting in an estimated 1.9 million new cases and about 935,000 deaths each year [1]. The burden of CRC varies greatly by geographic region, likely due to differences in lifestyle and socio-economic status [2]. China has the largest number of cases and CRC-related deaths as a result of its large population [3]. In 2016, it was estimated that about 408,000 new CRC cases and 195,600 CRC deaths occurred in China, giving it the second highest incidence and fourth highest mortality rate of all cancers [4]. While early screening and treatment have reduced the number of deaths [5], 25% of CRC patients are diagnosed with metastases, and 50% will go on to develop metastases [6], resulting in poor prognosis and a heavy disease burden.

Chemotherapy is the primary treatment for metastatic CRC (mCRC) [7]. However, chemotherapy alone is often limited by the lack of tumor cell selectivity, insufficient drug concentration in tumor tissues, systemic toxicity, and the appearance of drug-resistant tumor cells [8, 9]. According to the Pan-Asian adapted ESMO consensus guidelines for the management of patients with mCRC, the chemotherapy should be combinations of cytotoxic agents, in singlet, doublet, or combination chemotherapy along with appropriate targeted biological agents [10]. Over more than 10 years, the remission and survival conditions of mCRC patients have been revamped mainly because of development in prescribing conventional chemotherapy in combination with targeted agents. For example, clinical data indicated that the addition of bevacizumab to chemotherapy using fluorouracil, leucovorin, oxaliplatin, and irinotecan (FOLFOXIRI) prolonged median progression-free survival (PFS) from 9.7 to 12.1 months, and increased the overall response rate from 53% to 65% among the untreated mCRC patients [11]. A randomized controlled trial among Chinese patients with *RAS* wild-type mCRC found that first-line cetuximab combined with leucovorin, fluorouracil, and oxaliplatin (FOLFOX-4) improved the PFS (9.2 vs*.* 7.4 months), overall survival (OS) time (20.7 vs*.* 17.8 months) and overall response rate (61.1% vs*.* 39.5%) compared with FOLFOX-4 alone [12]. A meta-analysis demonstrated that aflibercept plus leucovorin calcium, fluorouracil, and irinotecan hydrochloride (FOLFIRI) showed better survival efficacies as a second-line treatment for mCRC compared with FOLFIRI alone [13]. In mCRC patients who had progressed following treatment with all approved standard drugs, both regorafenib and fruquintinib treatment offered substantial survival benefits. Compared with the placebo, patients treated with regorafenib or fruquintinib had prolonged OS and PFS [14–17].

Given the recognized clinical benefits of targeted cancer therapy, many countries and professional organizations have approved its use in clinical practice [18, 19]. In China, four targeted agents (bevacizumab, cetuximab, regorafenib, and fruquintinib) for mCRC were approved and listed in the medical insurance directory [19]. Bevacizumab, a recombinant humanized immunoglobulin monoclonal antibody that binds to and inhibits the activity of human vascular endothelial growth factor-A (VEGF-A), was approved for mCRC treatment in 2010 and is now used as a first- or second-line therapy in combination with chemotherapy [20, 21]. Cetuximab, an anti-epidermal growth factor receptor (EGFR) monoclonal antibody, was approved in China in December 2005 as a second- or third-line medication for mCRC and as a first-line therapy in combination with chemotherapy in September 2019 [12, 22]. Regorafenib, an oral multi-kinase inhibitor that blocks and inhibits the activity of multiple protein kinases including vascular endothelial growth factor receptors (VEGFRs), fibroblast growth factor receptor (FGFR), platelet-derived growth factor (PDGF)-α and -β, epidermal growth factor (EGF), angiotensin 2, and the Rapidly Accelerated Fibrosarcoma kinase pathway, was approved as the third-line therapy for mCRC in March 2017 [19, 23]. Fruquintinib, which inhibits VEGFR signaling was approved as the third-line therapy for mCRC in September 2018 [19, 24]. However, the use of targeted agents in real-world clinical practice has never been investigated. Thus, this study sought to explore the use of targeted therapy, identify potential barriers to its utilization, and identify factors associated with treatment initiation by Chinese mCRC patients. The goal was to provide reference data for clinicians and policymakers to prolong mCRC patient survival.

## Methods

### Study Design

This is a multicenter, cross-sectional, hospital-based clinical epidemiology study conducted in China, using stratified, multistage cluster sampling.

### Selection of Hospitals

The survey methodology was previously described [25]. In brief, China was stratified into seven geographic regions (East, North, South, Central, Northeast, Southwest, and Northwest) according to the traditional administrative district definition. The CRC disease burden differs by region [3]. During stage one, two cities from each geographical region were selected by simple random sampling. During stage two, one tertiary cancer hospital and/or one general hospital that represents the regional resources available to patients in each city were selected through convenience sampling based on their ability to provide CRC diagnosis, surgery, radiotherapy, chemotherapy, and routine follow-up care. A total of 19 tertiary hospitals (10 oncology and nine general hospitals) in 14 cities were involved in the study (Supplementary Figure S1).

### Selection of Patients

All mCRC patients at the selected hospitals were included if they were ≥18 years of age, had metastases and finished at least one cycle of chemotherapy, and provided informed consent. Patients were excluded if they had severe physical, cognitive, and/or verbal impairments that interfered with their ability to complete the questionnaire, or if their medical records on the use of targeted agents were unavailable.

### Data Collection

**FIGURE 1:**
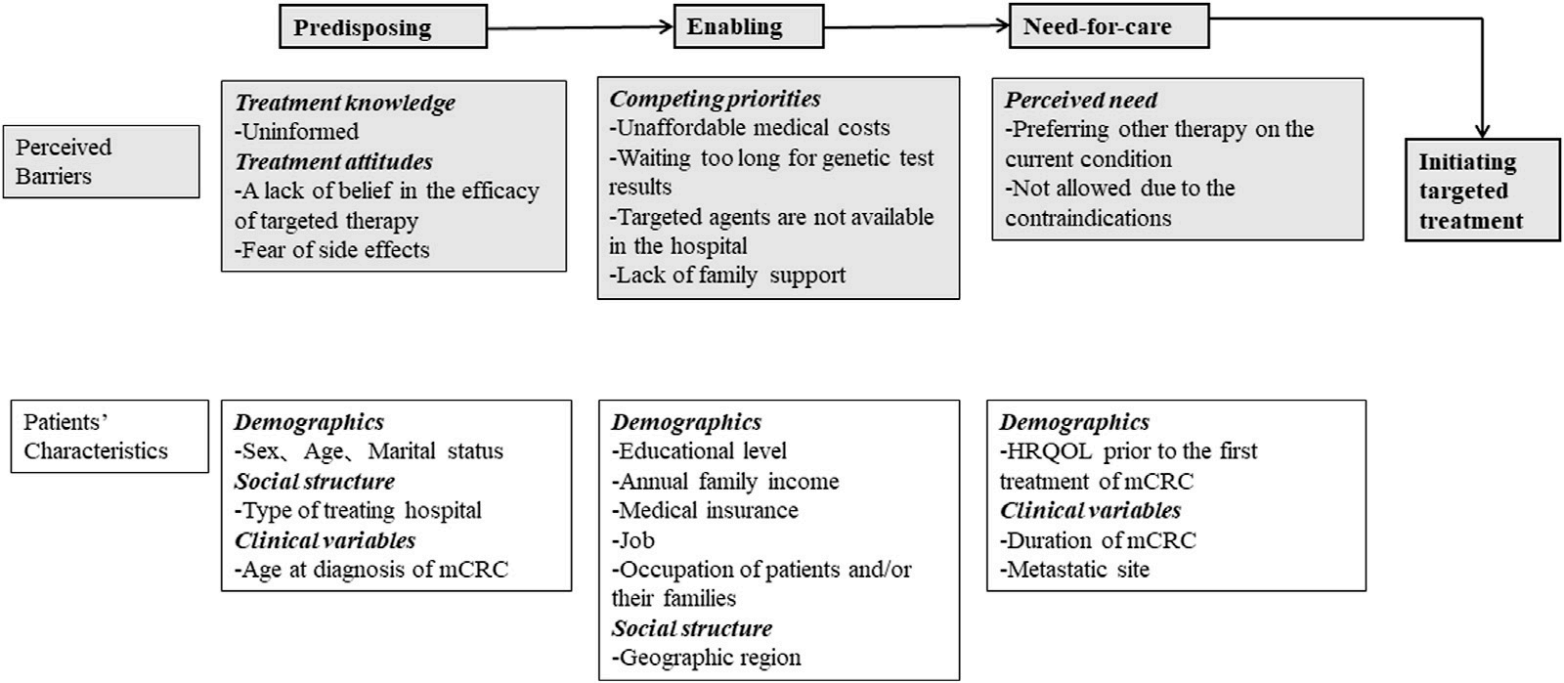
Framework of predisposing, enabling, and need factors for initiating targeted treatment. HRQOL, health-related quality of life; mCRC, metastatic colorectal cancer (China, 2023).

Well-trained clinical interviewers administered the survey to collect information on patient demographics, social structure, clinical results, utilization of targeted therapy, and barriers to treatment. The interviewers were clinicians or nurses from the selected hospitals, and members of the China Working Group on CRC Survey. Andersen’s behavior model for healthcare utilization was the framework used to identify the predisposing, enabling, and need-for-care factors affecting patient initiation of targeted therapy [26, 27]. Factors included in the model were self-reported perceived barriers and patient characteristics associated with targeted treatment (Figure 1).

### Study Measures

#### Utilization of Targeted Therapy and Self-Reported Barriers

Patient use of targeted treatment was collected through medical records. If patients had ever used a targeted agent, they were defined as “initiating targeted therapy,” otherwise, they were defined as “not initiating targeted therapy.” Of the patients who were “initiating targeted therapy,” the timing (first-, second-, and third-line) and types of targeted agents first used were obtained from medical records. Of those who were “not initiating targeted therapy,” related reasons or barriers to treatment were obtained through additional interviews administered by the clinical interviewers.

#### Patient Characteristics

Patient demographics, social structure, and clinical characteristics were collected from medical records and/or a standardized self-report questionnaire. The independent variables potentially associated with the initiation of targeted agents were selected using Andersen’s behavior model.

i. Predisposing factors included sex, marital status (married, single/divorced/widowed), and type of treating hospital (cancer, general), and age at diagnosis of mCRC (<65, ≥65 years of age).
ii. Enabling factors included educational level (0–6, 7–12, >12 years), annual family income (<50,000 CNY, 50,000–99,999 CNY, ≥ 100,000 CNY), medical insurance (none, private/public, private and public), job (unemployed, employed), occupation of the patients and/or their families (healthcare-related, non-healthcare-related), and geographic region (East, North, South, Central, Northeast, Southwest, and Northwest). If patients and/or their families worked in the pharmaceutical or medical device industries or were medical personnel (such as doctors, nurses, pharmacists, medical imagers, or medical examiners), their occupations were regarded as “healthcare-related,” otherwise, they were defined as “non-healthcare-related.”
iii. Need-for-care factors included health-related quality of life (HRQOL) prior to the first mCRC treatment, duration of mCRC (<10 and ≥10 months), and metastatic site (liver/lung, both liver and lung, outside liver/lung or systemic metastasis). HRQOL score was determined using the traditional Chinese Functional Assessment of Cancer Therapy-Colorectal (FACT-C, Version 4). It covers five function subscales (physical, social/family, emotional, functional, and colorectal cancer). Each item was ranked using a 5-point Likert scale (0–4). The total scores were calculated (ranging from 0 to 136) and classified as “poor” (total score ≤100) or “good” (total score >100) [28].

### Data Management and Quality Control

A non-identifiable paper-based questionnaire was used to manage individual participant data. Data obtained from the questionnaire were consistent with the participant self-report and medical record information. All collected data were double entered using Epidata software V.3.1 by two trained research assistants and an independent data administrator compared the entries to flag any discrepancies between the two datasets. The data were then checked using SAS software V.9.4 and any data queries were sent to the investigators to be resolved.

### Statistical Analysis

Descriptive analyses were used to report patient characteristics, distribution of the treatment timing (first-, second-, and third-line), the kinds of targeted agents used, and any identified barriers to treatment. Categorical variables were described as absolute frequencies and percentages, normally distributed continuous variables were shown using the mean and standard deviation (SD), and abnormally distributed continuous variables were described using the median and standard interquartile range (IQR, Q1–Q3).

Univariate and multivariate logistic regressions were conducted to identify the factors that correlated with initiating targeted therapy. Variables with a *p*-value < 0.1 in the univariate logistic regression model were candidates for multivariate regression analysis. A sequential series of multivariate regression models were developed to create the final multivariable model. Model 1 included predisposing factors, Model 2 superimposed enabling factors on Model 1, and Model 3 superimposed need-for-care factors on Model 2. The variables in the multivariate regression model were determined with the stepwise use of Akaike’s information criterion (AIC), and the model with the minimum AIC was adopted as the final model. Crude odds ratios (cOR) with 95% confidence intervals (CI) were reported for the univariate model, and adjusted odds ratio (aOR) with 95% CI were reported for the multivariate model. Sensitivity analysis was also conducted by limiting to patients with a mCRC duration of ≥10 months, to control for the impact of short-time duration on the use of targeted therapy. All statistical analyses were conducted using SAS V.9.4 software (SAS Institute Inc., Kerry, United States).

## Results

### Patient Characteristics

A total of 1,688 mCRC patients were recruited into the study. More than half (60.2%) were male, with an average age at diagnosis of 57.9 ± 11.57 years. Most patients were married (94.7%), had an education level of <12 years (82.4%), had an annual family income of <100,000 CNY (84.7%), and had medical insurance (98.6%). About half of the patients were treated at an oncology hospital (53.3%). The average HRQOL score prior to the initial treatment was 99.9 ± 20.96. The median duration of mCRC was 10.0 (3.0, 22.0). The liver or lung was the most frequent site of metastases (48.1%) (Table 1).

**TABLE 1:** Patient characteristics based on Andersen’s behavior model (China, 2023).

	Total (N, %)	East (N, %)	North (N, %)	South (N, %)	Central (N, %)	Northeast (N, %)	Southwest (N, %)	Northwest (N, %)
Predisposing factors								
Sex								
Male	1,017 (60.2)	309 (64.4)	98 (57.3)	135 (57.0)	113 (57.7)	90 (64.3)	210 (61.4)	62 (50.8)
Female	671 (39.8)	171 (35.6)	73 (42.7)	102 (43.0)	83 (42.3)	50 (35.7)	132 (38.6)	60 (49.2)
Marital status								
Married	1,598 (94.7)	460 (95.8)	164 (95.9)	222 (93.7)	183 (93.4)	132 (94.3)	321 (93.9)	116 (95.1)
Single/Divorced/Widow	90 (5.3)	20 (4.2)	7 (4.1)	15 (6.3)	13 (6.6)	8 (5.7)	21 (6.1)	6 (4.9)
Type of treating hospital								
Cancer hospital	900 (53.3)	213 (44.4)	68 (39.8)	120 (50.6)	128 (65.3)	55 (39.3)	241 (70.5)	75 (61.5)
General hospital	788 (46.7)	267 (55.6)	103 (60.2)	117 (49.4)	68 (34.7)	85 (60.7)	101 (29.5)	47 (38.5)
Age at mCRC diagnosis (years)								
<65	1,187 (70.3)	320 (66.7)	130 (76.0)	175 (73.8)	133 (67.9)	85 (60.7) (5.0)	252 (73.7)	92 (75.4)
≥ 65	501 (29.7)	160 (33.3)	41 (24.0)	62 (26.2)	63 (32.1)	55 (39.3)	90 (26.3)	30 (24.6)
Mean ± SD	57.9 ± 11.57	59.4 ± 10.86	58.5 ± 9.26	55.2 ± 13.65	58.2 ± 11.51	60.3 ± 12.01	56.7 ± 11.64	56.6 ± 10.81
Enabling factors								
Educational level (years)								
0–6	463 (27.5)	167 (34.9)	29 (17.0)	48 (20.3)	55 (28.1)	23 (16.4)	108 (31.6)	33 (27.0)
7–12	925 (54.9)	261 (54.5)	96 (56.1)	132 (55.9)	116 (59.2)	83 (59.3)	179 (52.3)	58 (47.5)
>12	298 (17.7)	51 (10.6)	46 (26.9)	56 (23.7)	25 (12.8)	34 (24.3)	55 (16.1)	31 (25.4)
Annual family income (CNY)								
<50,000	943 (55.9)	288 (60.0)	54 (31.6)	84 (35.4)	161 (82.1)	66 (47.1)	229 (67.0)	61 (50.0)
50,000–99,999	486 (28.8)	129 (26.9)	70 (40.9)	80 (33.8)	29 (14.8)	51 (36.4)	81 (23.7)	46 (37.7)
≥ 100,000	259 (15.3)	63 (13.1)	47 (27.5)	73 (30.8)	6 (3.1)	23 (16.4)	32 (9.4)	15 (12.3)
Medical insurance								
None	23 (1.4)	6 (1.3)	1 (0.6)	5 (2.1)	0 (0)	5 (3.6)	6 (1.8)	0 (0)
Private/Public	1,467 (86.9)	433 (90.2)	146 (85.4)	179 (75.5)	189 (96.4)	125 (89.3)	300 (87.7)	95 (77.9)
Private and Public	198 (11.7)	41 (8.5)	24 (14.0)	53 (22.4)	7 (3.6)	10 (7.1)	36 (10.5)	27 (22.1)
Job								
Unemployed	695 (41.2)	147 (30.6)	88 (51.5)	101 (42.6)	66 (33.7)	81 (57.9)	164 (48.0)	48 (39.3)
Employed	993 (58.8)	333 (69.4)	83 (48.5)	136 (57.4)	130 (66.3)	59 (42.1)	178 (52.0)	74 (60.7)
Patient and/or their family occupation								
Non-healthcare-related	1,469 (87.0)	426 (88.8)	152 (88.9)	216 (91.1)	170 (86.7)	129 (92.1)	284 (83.0)	92 (75.4)
Healthcare-related	219 (13.0)	54 (11.3)	19 (11.1)	21 (8.9)	26 (13.3)	11 (7.9)	58 (17.0)	30 (24.6)
Need-for-care factors								
HRQOL prior to the first mCRC treatment								
Poor	793 (47.0)	221 (46.0)	109 (63.7)	166 (70.0)	71 (36.2)	75 (53.6)	77 (22.5)	74 (60.7)
Good	895 (53.0)	259 (54.0)	62 (36.3)	71 (30.0)	125 (63.8)	65 (46.4)	265 (77.5)	48 (39.3)
Mean ± SD	99.9 ± 20.96	100.7 ± 21.39	92.4 ± 16.76	88.9 ± 18.42	103.5 ± 18.50	95.5 ± 23.08	112.6 ± 17.20	92.2 ± 19.58
Duration of mCRC (months)								
<10	839 (49.7)	241 (50.2)	72 (42.1)	129 (54.4)	94 (48.0)	61 (43.6)	179 (52.3)	63 (51.6)
≥ 10	849 (50.3)	239 (49.8)	99 (57.9)	108 (45.6)	102 (52.0)	79 (56.4)	163 (47.7)	59 (48.4)
Median (Q1, Q3)	10.0 (3.0, 22.0)	9.5 (3.0, 24.0)	12.0 (5.0, 23.0)	9.0 (4.0, 25.0)	10.0 (4.0, 21.0)	11.0 (3.0, 26.0)	8.0 (3.0, 18.0)	8.5 (4.0, 21.0)
Metastatic site								
Liver/lung	812 (48.1)	260 (54.2)	88 (51.5)	111 (46.8)	94 (48.0)	76 (54.3)	127 (37.1)	56 (45.9)
Liver and lung	190 (11.3)	39 (8.1)	27 (15.8)	26 (11.0)	19 (9.7)	23 (16.4)	44 (12.9)	12 (9.8)
Outside liver/lung or systemic metastasis	686 (40.6)	181 (37.7)	56 (32.7)	100 (42.2)	83 (42.3)	41 (29.3)	171 (50.0)	54 (44.3)
Initiated targeted therapy								
Yes	871 (51.6)	246 (51.3)	120 (70.2)	110 (46.4)	108 (55.1)	71 (50.7)	163 (47.7)	53 (43.4)
No	817 (48.4)	234 (48.8)	51 (29.8)	127 (53.6)	88 (44.9)	69 (49.3)	179 (52.3)	69 (56.6)

HRQOL, health-related quality of life; SD, standard deviation; CNY, Chinese Yuan

### Utilization of Targeted Therapy

**FIGURE 2:**
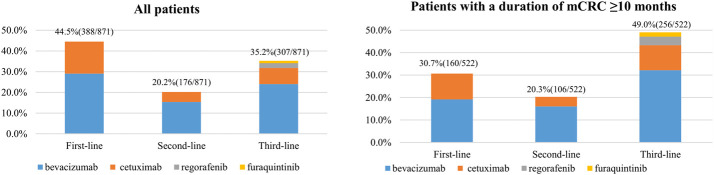
Distribution of the timing (first-line, second-line, and third-line) and kinds of targeted agents first used by metastatic CRC (mCRC) patients (China, 2023).

A total of 871 (51.6%) mCRC patients had ever initiated targeted therapy. Distribution of the timing (first-, second-, and third-line) and the kinds of targeted agents first used by mCRC patients are shown in Figure 2A. Of the patients who initiated targeted therapy, 44.5% (388/871), 20.2% (176/871), and 35.2% (307/871) began the medication as a first-, second-, and third-line treatment, respectively. Of those who initiated targeted therapy as the first-line treatment, 65.2% (253/388) and 34.8% (135/388) used bevacizumab and cetuximab, respectively. Of those who first initiated targeted therapy as the second-line treatment, 76.1% (134/176) and 23.9% (42/176) used bevacizumab and cetuximab, respectively. Of those who first initiated targeted therapy as the third-line treatment, 68.1% (209/307), 22.1% (68/307), 6.5% (20/307), and 3.3% (10/307) used bevacizumab, cetuximab, regorafenib, and fruquintinib, respectively. Overall, bevacizumab was the most frequently used targeted agent (68.4%, 596/871), followed by cetuximab (28.1%, 245/871), regorafenib (2.3%, 20/871), and fruquintinib (1.1%, 10/871). These drugs were similarly distributed among patients with an mCRC duration of ≥10 months (Figure 2B).

### Self-Reported Barriers to the Initiation of Targeted Therapy

**FIGURE 3:**
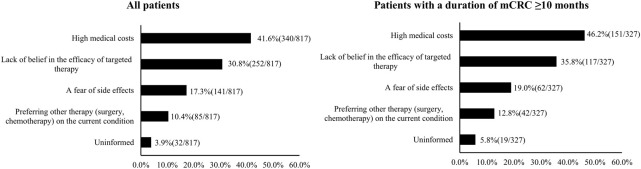
Barriers to the initiation of targeted therapy by metastatic CRC (mCRC) patients (China, 2023).

Self-reported barriers to the initiation of targeted therapy are shown in Figure 3A. The most common barriers included high medical costs (41.6%, 340/817), lack of belief in the efficacy of targeted therapy (30.8%, 252/817), and a fear of side effects (17.3%, 141/817). The distribution of self-reported barriers was similar among patients with an mCRC duration ≥10 months (Figure 3B).

### Factors Associated With Initiating Targeted Treatment

The type of hospital, age at mCRC diagnosis, educational level, annual family income, medical insurance, geographic region, HRQOL score prior to first treatment, duration of mCRC, and metastatic site were variables potentially associated with the initiation of targeted therapy and were included in a sequential series of multivariate models (Table 2).

**TABLE 2:** Univariate analysis of initiating targeted therapy among mCRC (China, 2023).

	Initiating targeted therapy		Univariate model	
	Yes (*n* = 871)	No (*n* = 817)	cOR (95% CI)	*P*
Predisposing factors				
Sex				
Male	521 (51.2)	496 (48.8)	Reference	
Female	350 (52.2)	321 (47.8)	1.038 (0.854–1.261)	0.708
Marital status				
Married	830 (51.9)	768 (48.1)	Reference	
Single/Divorced/Widow	41 (45.6)	49 (54.4)	0.774 (0.505–1.186)	0.240
Type of treating hospital				
Cancer hospital	510 (56.7)	390 (43.3)	Reference	
General hospital	361 (45.8)	427 (54.2)	0.646 (0.533–0.783)	<0.001
Age at mCRC diagnosis (years)				
<65	651 (54.8)	536 (45.2)	Reference	
≥ 65	220 (43.9)	281 (56.1)	0.645 (0.522–0.795)	<0.001
Enabling factors				
Educational level (years)				
0–6	197 (42.5)	266 (57.5)	0.506 (0.376–0.680)	<0.001
7–12	497 (53.7)	428 (46.3)	0.794 (0.609–1.034)	0.087
>12	177 (59.4)	121 (40.6)	Reference	
Annual family income (CNY)				
<50,000	455 (48.3)	488 (51.7)	0.549 (0.413–0.728)	<0.001
50,000–99,999	253 (52.1)	233 (47.9)	0.639 (0.469–0.870)	0.004
≥ 100,000	163 (62.9)	96 (37.1)	Reference	
Medical insurance				
None	5 (21.7)	18 (78.3)	0.192 (0.068–0.538)	0.002
Private/Public	749 (51.1)	718 (48.9)	0.722 (0.530–0.976)	0.034
Private and Public	117 (59.1)	81 (40.9)	Reference	
Job				
Un-employed	374 (53.8)	321 (46.2)	Reference	
Employed	497 (50.1)	496 (49.9)	0.860 (0.708–1.045)	0.128
Patient and/or their family occupation				
Non-healthcare-related	751 (51.1)	718 (48.9)	0.863 (0.649–1.148)	0.312
Healthcare-related	120 (54.8)	99 (45.2)	Reference	
Geographic region				
East	246 (51.3)	234 (48.8)	0.857 (0.614–1.196)	0.363
North	120 (70.2)	51 (29.8)	1.916 (1.244–2.952)	0.003
South	110 (46.4)	127 (53.6)	0.706 (0.483–1.032)	0.072
Central	108 (55.1)	88 (44.9)	Reference	
Northeast	71 (50.7)	69 (49.3)	0.838 (0.543–1.295)	0.427
Southwest	163 (47.7)	179 (52.3)	0.742 (0.522–1.056)	0.097
Northwest	53 (43.4)	69 (56.6)	0.626 (0.397–0.987)	0.044
Need-for-care factors				
HRQOL prior to the first mCRC treatment				
Poor	369 (46.5)	424 (53.5)	0.681 (0.560–0.825)	<0.001
Good	502 (56.1)	393 (43.9)	Reference	
Duration of mCRC (months)				
<10	349 (41.6)	490 (58.4)	0.446 (0.367–0.54)	<0.001
≥ 10	522 (61.5)	327 (38.5)	Reference	
Metastatic site				
Liver/lung	429 (52.8)	383 (47.2)	Reference	
Liver and lung	123 (64.7)	67 (35.3)	1.634 (1.180–2.274)	0.003
Outside liver/lung or systemic metastasis	319 (46.5)	367 (53.5)	0.776 (0.632–0.951)	0.015

cOR, crude odds ratio; CI, confidence interval; CNY, Chinese Yuan; HRQOL, health-related quality of life.

Model 3 was the final multivariate regression model with the lowest AIC. In this final model, patients who were treated in the general hospital (aOR: 0.580, 95% CI: 0.466–0.721), were diagnosed with mCRC at ≥65 years of age (aOR: 0.780, 95% CI: 0.620–0.982), had <7 years of education (aOR: 0.703, 95% CI: 0.494–0.999), had an annual family income <50,000 CNY (aOR: 0.707, 95% CI: 0.501–0.996), had no medical insurance (aOR: 0.203, 95% CI: 0.069–0.593), were located in Southwest China (aOR: 0.684, 95% CI: 0.470–0.994) or Northwest China (aOR: 0.617, 95% CI: 0.237–0.997), had a poor HRQOL score prior to the first mCRC treatment (aOR: 0.729, 95% CI: 0.586–0.907), had an mCRC duration <10 months (aOR: 0.469, 95% CI: 0.382–0.576), or had metastases outside liver/lung or systemic metastasis (aOR: 0.753, 95% CI: 0.606–0.936) were less likely to initiate targeted treatment. Patients who lived in North China (aOR: 1.992, 95% CI: 1.239–3.202), and those with both liver and lung metastases (aOR: 1.614, 95% CI: 1.136–2.295) were more likely to initiate targeted therapy (Table 3).

**TABLE 3:** Multivariate analysis of initiating targeted therapy among mCRC (China, 2023).

	Model 1		Model 2		Model 3	
	aOR (95% CI)	*P*	aOR (95% CI)	*P*	aOR (95% CI)	*P*
Predisposing factors						
Type of treating hospital						
Cancer hospital	Reference		Reference		Reference	
General hospital	0.683 (0.562–0.830)	<0.001	0.608 (0.494–0.748)	<0.001	0.580 (0.466–0.721)	<0.001
Age at mCRC diagnosis (years)						
<65	Reference		Reference		Reference	
≥ 65	0.681 (0.550–0.843)	<0.001	0.745 (0.597–0.931)	0.010	0.780 (0.620–0.982)	0.034
Enabling factors						
Educational level (years)						
0–6			0.703 (0.499–0.990)	0.043	0.703 (0.494–0.999)	0.049
7–12			0.978 (0.732–1.307)	0.882	0.978 (0.726–1.317)	0.882
>12			Reference		Reference	
Annual family income (CNY)						
<50,000			0.674 (0.485–0.938)	0.019	0.707 (0.501–0.996)	0.049
50,000–99,999			0.698 (0.504–0.969)	0.032	0.731 (0.522–1.022)	0.067
≥ 100,000			Reference		Reference	
Medical insurance						
None			0.191 (0.067–0.548)	0.002	0.203 (0.069–0.593)	0.004
Private/Public			0.753 (0.545–1.042)	0.087	0.775 (0.555–1.082)	0.135
Private and Public			Reference		Reference	
Geographic region						
East			0.938 (0.663–1.328)	0.719	0.986 (0.688–1.413)	0.939
North			1.875 (1.187–2.961)	0.007	1.992 (1.239–3.202)	0.004
South			0.625 (0.416–0.938)	0.023	0.733 (0.479–1.123)	0.153
Central			Reference		Reference	
Northeast			0.896 (0.568–1.413)	0.637	0.851 (0.532–1.362)	0.502
Southwest			0.693 (0.482–0.995)	0.047	0.684 (0.470–0.994)	0.047
Northwest			0.553 (0.344–0.888)	0.014	0.617 (0.237–0.997)	0.043
Need-for-care factors						
HRQOL prior to the first mCRC treatment						
Poor					0.729 (0.586–0.907)	0.005
Good					Reference	
Duration of mCRC (months)						
<12					0.469 (0.382–0.576)	<0.001
≥ 12					Reference	
Metastatic site						
Liver/lung					Reference	
Liver and lung					1.614 (1.136–2.295)	0.008
Outside liver/lung or systemic metastasis					0.753 (0.606–0.936)	0.011
AIC	2308.36		2258.36		2178.60	

The variables in the multivariate regression model were determined through step-wise method.

Model 1 included predisposing factors; Model 2 superimposed enabling factors on Model 1; Model 3 superimposed need-for-care factors on Model 2.

aOR, adjusted odds ratio; CI, confidence interval; CNY, Chinese Yuan; HRQOL, health-related quality of life; AIC, Akaike’s information criterion.

After limiting the analysis to patients with an mCRC duration ≥ 10 months, the factors associated with initiating targeted therapy remained mostly similar, except for annual family income and medical insurance due to low power (Supplementary Tables S1, S2).

## Discussion

This study is the first to provide real-world evidence in support of the use of targeted treatment for mCRC patients in China. The findings illustrated that 51.6% of mCRC patients had ever initiated targeted therapy, one-third of whom first initiated targeted therapy as the third-line treatment. Bevacizumab was the most commonly used medication. High medical cost, lack of belief in the efficacy of targeted therapy and a fear of side effects were the most frequently reported barriers to the initiation of targeted therapy.

While targeted agents have been approved for >10 years, and are recommended by Chinese clinical guidelines, use remains lower than in developed countries. In the United States, for example, >70% of CRC patients with stage IV disease have ever received targeted therapy [29]. The high medical cost was the predominant barrier against initiating targeted agents in this study. In China, the cost for a 1 month’s supply of bevacizumab, cetuximab, regorafenib, fruquintinib is about 9,500 CNY [30], 15,000 CNY [31], 15,000 CNY, and 5,500 CNY [32], respectively. However, more than half of mCRC patients have an annual family income of <50,000 CNY to cover their daily spending needs. While all the targeted agents were listed in the medical insurance directory, and 98.6% of the patients had insurance, the reimbursement rate was limited. In addition, particular limits were added to the cost of treatment during hospitalization. Thus, a cost-reduction strategy may be the first step to facilitate the use of targeted therapy.

Concern about the efficacy and safety of targeted therapy was also a common barrier to treatment initiation. More than one-third of mCRC patients delayed targeted treatment until it became the third-line option, despite studies illustrating that bevacizumab and cetuximab are effective as first- or second-line treatments at improving survival and quality of life [10, 11]. In addition, while regorafenib and fruquintinib have been recommended as the standard third-line mCRC drugs in China since 2017 [19], most patients in the current study still used bevacizumab or cetuximab, suggesting that the misuse of targeted agents is common. Thus, there is a need to improve public awareness of targeted treatments and to strengthen prescriber knowledge of clinical guidelines to promote the timely and correct application of targeted drugs.

Bevacizumab was most frequently used as a first- or second-line drug. In addition to drug cost, the affordability and availability of genetic biomarker testing may also influence the choice of targeted therapy. More than half of advanced CRC patients in the current study had never received biomarker testing due to its high price [33]. Bevacizumab may be an appealing drug choice because it targets VEGF and does not require genetic biomarker testing [21]. However, precision medicine dictates that target drugs are most effective if a predictive biomarker is available and agents that are exempt from biomarker testing should not be selected for use. Biomarkers are also recognized as predictors of targeted drugs and drug reactions and are thus recommended for the selection of personalized therapies by some leading guidelines [6, 34, 35]. For example, cetuximab is associated with a longer OS, a higher objective response rate, a higher complete response, and a greater median depth of response than bevacizumab for the treatment of *RAS* and *BRAF* wild-type mCRC [36]. Therefore, policymakers should implement specific strategies, including reducing the cost of testing for key biomarkers and ensuring that they are covered by insurance, to facilitate the use of biomarker tests.

Consistent with patient self-reported barriers to initiating targeted therapy, patients with less education, low annual family income, and no medical insurance were less likely to start targeted treatment. Education level was positively associated with CRC awareness and knowledge [37], thus, less educated patients may not be convinced that targeted therapy is effective. Patients with a low annual family income or no medical insurance may also refuse targeted therapy due to its high expense. Previous studies indicate that elderly patients have limited heath care utilization because they are likely to have less education, lower income, and receive less family support [38, 39]. The current study also found that older patients had a lower probability of using targeted therapy, suggesting that more attention should be paid to this subgroup to ensure equitable cancer care utilization. Slight differences in the use of targeted therapy were also shown by geographic region. Patients who lived in North China had the highest likelihood of initiating targeted therapy, while those living in Southwest or Northwest China had the lowest. North China has a stronger economy, culture, and healthcare system than Southwest and Northwest China, indicating that these factors may be positively associated with the use of targeted therapy. Beijing, which was randomly selected for this survey, is the city with the highest economy, culture, and healthcare services in North China. Thus, this may also have contributed to the high number of patients initiating targeted therapy in this region. These findings highlight that less developed regions should be prioritized for interventions and health education to improve access to targeted treatment.

Patient clinical characteristics were also associated with the likelihood that they would initiate targeted therapy. Those with a shorter duration of mCRC were less likely to initiate targeted therapy. This may be because symptoms were often relatively mild in these patients, suggesting that they may have a higher level of medication hesitation. Thus, more attention and care should be paid to this population. Importantly, patients with more severe disease, including those with low HRQOL scores prior to treatment and those with metastases outside the liver/lung or systemic metastasis, were also less likely to initiate targeted therapy. It is possible that supportive care, including pain management, nutritional support, and psychological intervention may be a better choice for these patients [40, 41]. These findings highlight the need to address targeted treatment hesitation to prevent mCRC from developing to a point at which targeted therapy is no longer an effective option.

Interestingly, patients who were treated in a general hospital were less likely to initiate targeted therapy than those who received treatment in an oncology hospital. This may be because more patients with underlying diseases (such as diabetes, hypertension, and renal insufficiency) or poor organ function that contribute to a poor HRQOL prefer to be treated in a general hospital. Indeed, this study found that patients treated in general hospitals had a lower HRQOL score than those treated in oncology hospitals (94.4 ± 23.12 vs. 104.8 ± 17.42; *p* < 0.001). Additional data is needed to further assess this hypothesis.

The strengths of this study included its large sample size, multi-center survey nature and hospital-based representative sampling. To our knowledge, this is the first geographically representative study that includes a large number (>1,600) of mCRC patients in China, allowing for a reliable and robust analysis. This study provides real-world evidence for clinicians and policymakers to design interventions that promote the use of targeted therapy and reduce CRC-related mortality.

This study also had several limitations. First, because it is a cross-sectional survey study, causal relationships between patient characteristics and the initiation of targeted treatment cannot be firmly established. Second, the self-reported data may be subject to recall and social desirability biases. Third, since the patients volunteered to enroll, their characteristics may differ from those who did not chose to participate in the study. Finally, only those patients who finished at least the first cycle of chemotherapy were included. The short treatment interval might underestimate the rate of initiating targeted therapy since some patients may delay the use of targeted agents.

### Conclusion

This study highlights the limited use of targeted therapy and the frequency of delayed treatment using targeted agents for mCRC patients in China. Bevacizumab was the most frequently used drug. The common barriers to initiating targeted treatment were the high medical cost, the lack of belief in the efficacy of targeted treatment, and a fear of side effects. Reducing the cost of treatment and providing interventional education to improve public awareness are recommended to facilitate the use of targeted therapy.

## Data Availability

The raw data supporting the conclusion of this article will be made available by the authors, without undue reservation.
