# Supplementary material for: Real-World Utilization, Barriers, and Factors Associated With the Targeted Treatment of Metastatic Colorectal Cancer Patients in China: A Multi-Center, Hospital-Based Survey Study
Source: Int J Public Health. 2023 Jul 3;68:1606091. doi: 10.3389/ijph.2023.1606091 (PMC10351535; doi:10.3389/ijph.2023.1606091)
Supplement: Supplementary file 1 [file Table1.DOCX]

**Table S1** Univariate analysis of initiating targeted therapy among patients who had a duration of mCRC $\geq$10 months in China

|  | Initiating targeted therapy | |  | | Univariate model | |  |
| --- | --- | --- | --- | --- | --- | --- | --- |
|  | Yes (n=522) | No (n=327) |  | | cOR (95% CI) | | *P* |
| ***Predisposing factors*** |  |  |  | |  | |  |
| **Sex** |  |  |  | |  | |  |
| Male | 313 (62.0) | 192 (38.0) |  | | Reference | |  |
| Female | 209 (60.8) | 135 (39.2) |  | | 0.949 (0.716-1.258) | | 0.718 |
| **Marital status** |  |  |  | |  | |  |
| Married | 503 (61.9) | 309 (38.1) |  | | Reference | |  |
| Single/Divorced/Widow | 19 (51.4) | 18 (48.6) |  | | 0.648 (0.335-1.25) | | 0.198 |
| **Type of treating hospital** |  |  |  | |  | |  |
| Cancer hospital | 296 (68.0) | 139 (32.0) |  | | Reference | |  |
| general hospital | 226 (54.6) | 188 (45.4) |  | | 0.564 (0.426-0.746) | | <0.001 |
| **Age at mCRC diagnosis** |  |  |  | |  | |  |
| <65 | 410 (65.5) | 216 (34.5) |  | | Reference | |  |
| $\geq$65 | 112 (50.2) | 111 (49.8) |  | | 0.534 (0.391-0.728) | | <0.001 |
| ***Enabling factors*** |  |  |  | |  | |  |
| **Educational level (years)** |  |  |  | |  | |  |
| 0-6 | 116 (53.0) | 103 (47.0) |  | | 0.444 (0.289-0.683) | | <0.001 |
| 7-12 | 287 (62.1) | 175 (37.9) |  | | 0.648 (0.440-0.953) | | 0.027 |
| >12 | 119 (71.7) | 47 (28.3) |  | | Reference | |  |
| **Annual family income (CNY)** |  |  |  | |  | |  |
| < 50,000 | 150 (58.8) | 105 (41.2) |  | | 0.553 (0.356-0.85) | | 0.008 |
| 50,000-99,999 | 266 (59.5) | 181 (40.5) |  | | 0.569 (0.378-0.85) | | 0.006 |
| $\geq$100,000 | 106 (72.1) | 41 (27.9) |  | | Reference | |  |
| **Medical insurance** |  |  |  | |  | |  |
| None | 4 (40.0) | 6 (60.0) |  | | 0.311 (0.082-1.173) | | 0.085 |
| Private/Public | 443 (60.8) | 286 (39.2) |  | | 0.723 (0.471-1.108) | | 0.137 |
| Private and Public | 75 (68.2) | 35 (31.8) |  | | Reference | |  |
| **Job** |  |  |  | |  | |  |
| Un-employed | 228 (61.6) | 142 (38.4) |  | | Reference | |  |
| Employed | 294 (61.4) | 185 (38.6) |  | | 0.989 (0.748-1.308) | | 0.942 |
| **Patient and/or their family occupation** |  |  |  | |  | |  |
| Nonhealthcare-related | 453 (60.7) | 293 (39.3) |  | | 0.762 (0.492-1.178) | | 0.222 |
| Healthcare-related | 69 (67.0) | 34 (33.0) |  | | Reference | |  |
| **Geographic region** |  |  |  |  | |  | |
| East | 153 (64.0) | 86 (36.0) |  | 1.245 (0.775-2.002) | | 0.365 | |
| North | 77 (77.8) | 22 (22.2) |  | 2.450 (1.323-4.538) | | 0.004 | |
| South | 64 (59.3) | 44 (40.7) |  | 1.018 (0.587-1.765) | | 0.949 | |
| Central | 60 (58.8) | 42 (41.2) |  | Reference | |  | |
| Northeast | 42 (53.2) | 37 (46.8) |  | 0.795 (0.439-1.437) | | 0.447 | |
| Southwest | 90 (55.2) | 73 (44.8) |  | 0.863 (0.523-1.424) | | 0.564 | |
| Northwest | 36 (61.0) | 23 (39.0) |  | 1.096 (0.569-2.110) | | 0.785 | |
| ***Need-for-care factors*** |  |  |  | |  | |  |
| **HRQOL prior to the first mCRC treatment** |  |  |  | |  | |  |
| Poor | 212 (55.9) | 167 (44.1) |  | | 0.655 (0.495-0.865) | | 0.003 |
| Good | 310 (66.0) | 160 (34.0) |  | | Reference | |  |
| **Metastatic site** |  |  |  | |  | |  |
| Liver/lung | 254 (62.0) | 156 (38.0) |  | | Reference | |  |
| Liver and lung | 85 (70.8) | 35 (29.2) |  | | 1.491 (0.959-2.318) | | 0.076 |
| Outside liver/lung or systemic metastasis | 183 (57.4) | 136 (42.6) |  | | 0.826 (0.613-1.113) | | 0.210 |

^cOR: crude odds ratio; CI: Confidence Interval; CNY, Chinese Yuan; HRQOL, health-related quality of life^
