# Supplementary figures and images for: Real-World Utilization, Barriers, and Factors Associated With the Targeted Treatment of Metastatic Colorectal Cancer Patients in China: A Multi-Center, Hospital-Based Survey Study
Source: Int J Public Health. 2023 Jul 3;68:1606091. doi: 10.3389/ijph.2023.1606091 (PMC10351535; doi:10.3389/ijph.2023.1606091)

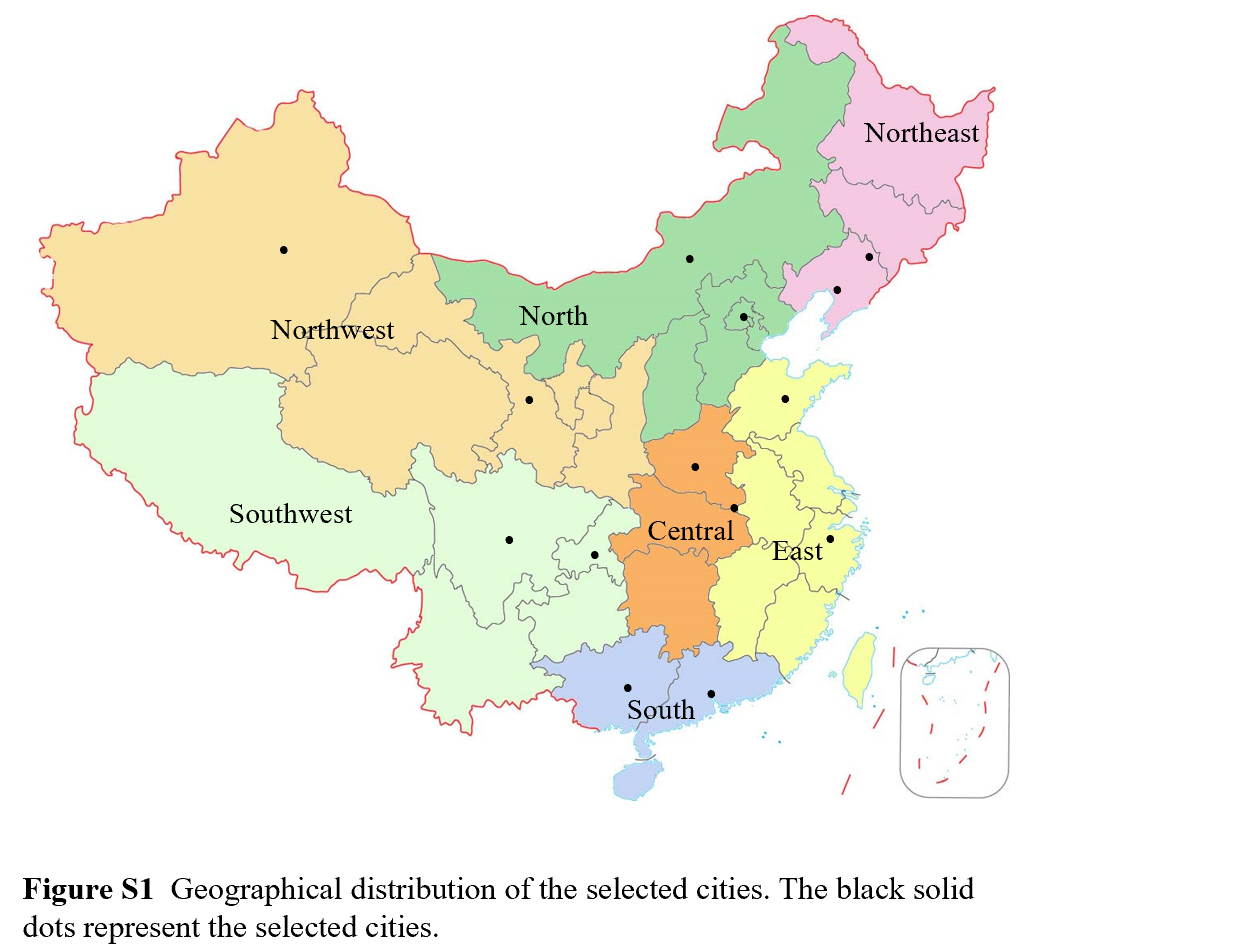

Supplement: Supplementary file 2 [file Image1.TIF]
