# Supplementary material for: Real-World Utilization, Barriers, and Factors Associated With the Targeted Treatment of Metastatic Colorectal Cancer Patients in China: A Multi-Center, Hospital-Based Survey Study
Source: Int J Public Health. 2023 Jul 3;68:1606091. doi: 10.3389/ijph.2023.1606091 (PMC10351535; doi:10.3389/ijph.2023.1606091)
Supplement: Supplementary file 3 [file Table2.DOCX]

**Table S2** Multivariate analysis of initiating targeted therapy among patients who had a duration of mCRC $\geq$10 months in China

|  | Model 1 | |  | Model 2 | |  | Model 3 | |
| --- | --- | --- | --- | --- | --- | --- | --- | --- |
|  | aOR (95% CI) | *P* |  | aOR (95% CI) | *P* |  | aOR (95% CI) | *P* |
| ***Predisposing factors*** |  |  |  |  |  |  |  |  |
| **Type of treating hospital** |  |  |  |  |  |  |  |  |
| Cancer hospital | Reference |  |  | Reference |  |  | Reference |  |
| General hospital | 0.610 (0.460-0.811) | <0.001 |  | 0.585 (0.435-0.788) | <0.001 |  | 0.611 (0.450-0.830) | <0.001 |
| ***Enabling factors*** |  |  |  |  |  |  |  |  |
| **Educational level (years)** |  |  |  |  |  |  |  |  |
| 0-6 |  |  |  | 0.513 (0.325-0.807) | 0.004 |  | 0.503 (0.318-0.796) | 0.003 |
| 7-12 |  |  |  | 0.703 (0.471-1.049) | 0.084 |  | 0.684 (0.457-1.024) | 0.065 |
| >12 |  |  |  | Reference |  |  | Reference |  |
| **Annual family income (CNY)** |  |  |  |  |  |  |  |  |
| < 50,000 |  |  |  |  |  |  |  |  |
| 50,000-99,999 |  |  |  |  |  |  |  |  |
| $\geq$100,000 |  |  |  |  |  |  |  |  |
| **Medical insurance** |  |  |  |  |  |  |  |  |
| None |  |  |  |  |  |  |  |  |
| Private/Public |  |  |  |  |  |  |  |  |
| Private and Public |  |  |  |  |  |  |  |  |
| **Geographic region** |  |  |  |  |  |  |  |  |
| East |  |  |  | 1.419 (0.867-2.321) | 0.164 |  | 1.519 (0.917-2.517) | 0.104 |
| North |  |  |  | 2.618 (1.384-4.952) | 0.003 |  | 3.053 (1.586-5.875) | <0.001 |
| South |  |  |  | 0.981 (0.554-1.740) | 0.949 |  | 1.164 (0.645-2.098) | 0.614 |
| Central |  |  |  | Reference |  |  | Reference |  |
| Northeast |  |  |  | 0.932 (0.503-1.729) | 0.824 |  | 0.942 (0.503-1.765) | 0.853 |
| Southwest |  |  |  | 0.866 (0.517-1.452) | 0.585 |  | 0.830 (0.489-1.409) | 0.491 |
| Northwest |  |  |  | 1.067 (0.543-2.097) | 0.852 |  | 1.221 (0.613-2.432) | 0.570 |
| ***Need-for-care factors*** |  |  |  |  |  |  |  |  |
| **HRQOL prior to the first mCRC treatment** |  |  |  |  |  |  |  |  |
| Poor |  |  |  |  |  |  | 0.606 (0.443-0.830) | 0.002 |
| Good |  |  |  |  |  |  | Reference |  |
| **Metastatic site** |  |  |  |  |  |  |  |  |
| Liver/lung |  |  |  |  |  |  | Reference |  |
| Liver and lung |  |  |  |  |  |  | 1.583 (1.002-2.508) | 0.049 |
| Outside liver/lung or systemic metastasis |  |  |  |  |  |  | 0.783 (0.570-1.075) | 0.130 |
| **AIC** | 1106.64 | |  | 1094.13 | |  | 1081.78 | |

^The variables in the multivariate regression model were determined through step-wise method.^

^Model 1 included predisposing factors; Model 2 superimposed enabling factors on Model 1; Model 3 superimposed need-for-care factors on Model 2.^

^aOR: adjusted odds ratio; CI: Confidence Interval; CNY: Chinese Yuan; HRQOL: health-related quality of life; AIC: Akaike’s information criterion.^
